# Repurposing Kinase Inhibitor Bay 11-7085 to Combat *Staphylococcus aureus* and *Candida albicans* Biofilms

**DOI:** 10.3389/fphar.2021.675300

**Published:** 2021-05-05

**Authors:** Iliana E. Escobar, Fernanda Cristina Possamai Rossatto, Soo Min Kim, Min Hee Kang, Wooseong Kim, Eleftherios Mylonakis

**Affiliations:** ^1^ Infectious Diseases Division, Department of Medicine, Warren Alpert Medical School of Brown University, Rhode Island Hospital, Providence, RI, United States; ^2^ Laboratory of Biofilms and Alternative Models, Federal University of Health Sciences of Porto Alegre, Porto Alegre, Brazil; ^3^ College of Pharmacy, Graduate School of Pharmaceutical Sciences, Ewha Womans University, Seoul, Republic of Korea

**Keywords:** biofilms, *Staphylococcus aureus*, *Candida albicans*, antibiotic resistance, Bay 11-7085, *C. elegans* high through-put screening

## Abstract

*Staphylococcus aureus* and *Candida* spp. are commonly linked with topical biofilm-associated infections such as those found on chronic wounds. These biofilms are notoriously difficult to treat, highlighting the grave need to discover and study new broad-spectrum agents to combat associated infections. Here we report that the kinase inhibitor Bay 11-7085 exhibited bactericidal activity against multidrug-resistant *S. aureus* with a minimum inhibitory concentration (MIC) of 4 μg/ml. In addition, *S. aureus* strain MW2 did not acquire resistance to antibiotic pressure. Furthermore, Bay 11-7085 exhibited potency against *Candida albicans* and the emerging pathogen *Candida auris* with a MIC of 0.5–1 μg/ml. Bay 11-7085 partially inhibited and eradicated biofilm formation of various pathogens, such as VRSA (vancomycin-resistant *S. aureus*), as well as antifungal-resistant *Candida* spp. isolates. Notably, Bay 11-7085 partially inhibited initial cell attachment and formation of a VRSA-*C. albicans* polymicrobial biofilm *in vitro*. In contrast to *C. albicans*, inhibition of VRSA biofilm was linked to initial cell attachment independent of its bactericidal activity. Finally, Bay 11-7085 was effective *in vivo* at increasing the lifespan of *C. elegans* during an *S. aureus* and a *C. albicans* infection. Our work proposes kinase inhibitor Bay 11-7085 as a potential compound capable of combating biofilms associated with primary multidrug-resistant bacteria and yeast pathogens associated with wound infections.

## Introduction

With the diminished efforts focusing on discovering new antimicrobial agents, antibiotic resistance has been a persistent problem hindering the treatment of bacterial and fungal infections (Munita and Arias, 2016). In addition to spontaneous mutations, antimicrobial resistance is augmented through a pathogens transition from a planktonic state to a biofilm (Stewart, 2002). Biofilms are an organization of microorganisms adherent to themselves and other surfaces which produce an extracellular matrix (ECM) (Moormeier and Bayles, 2017; Cavalheiro and Teixeira, 2018) and confer antimicrobial tolerance to their resident microbes through three main mechanisms: adaptive stress, low metabolic cells, and physical blockage of antimicrobial penetration by the ECM (Stewart, 2002). Thus, cells within biofilms are known to have up to 10,000-fold higher tolerance to antimicrobial agents (Sharma et al., 2019).

Although biofilm formations exact mechanism can differ from species to species, most microorganisms share a common scaffold of multiple stages of maturation (Moormeier and Bayles, 2017). The first stage is initial cell attachment, which serves as the microbial network foundation to begin signaling and building the ECM (Moormeier and Bayles, 2017; Cavalheiro and Teixeira, 2018). Then, during multiplication and maturation, attached cells start to divide and form colonies that commence interlocking and consequently fortify the ECM (Moormeier and Bayles, 2017; Cavalheiro and Teixeira, 2018). Lastly, through detachment and dispersal, biofilms begin to spread by releasing cells from within the ECM and initiating the cycle once again (Moormeier and Bayles, 2017; Cavalheiro and Teixeira, 2018). Recent studies have evaluated strategies to combat biofilm-associated infections by targeting different stages of biofilm formation. For example, aryl rhodanines or calcium chelators have shown some success at inhibiting biofilm initial cell attachment (Chung and Toh, 2014). Metallic silver, silver ions, and silver nanoparticles show efficacy as a broad anti-biofilm effective against Gram-negative, Gram-positive, and fungal pathogens (Chung and Toh, 2014). In addition, molecules such a *cis*-2-decenoic acid, the peptide dispersin B, or the quorum-sensing inhibitor RNAIII-inhibiting peptide (RIP) have shown efficacy at disrupting fully mature biofilm *in vivo* (Chung and Toh, 2014). However, only silver is an FDA-approved molecule, further emphasizing the need for new anti-biofilm drug targets.

Clinically, 90% of chronic wounds are associated with biofilms and persistent inflammation (Attinger and Wolcott, 2012). There are multiple stages of wound healing. However, chronic wounds fail to cycle through each phase in less than 3 months or more due to ongoing inflammation and failed antibiotic therapy (Bowers and Franco, 2020). Chronic wounds can include venous, diabetic, or pressure ulcers (Bowers and Franco, 2020). In addition, chronic wounds pose a significant financial burden to our health care system. In 2014, Nussbaum et al., estimated a total cost burden of $28–96.8 billion dollars (Nussbaum et al., 2018). Furthermore, chronic wounds may lead to a deteriorated quality of life followed by depression and isolation (Beitz and Goldberg, 2005). Other biofilm-associated wounds include those caused by extreme burns or traumatic injury, both of which are difficult to treat and heal (Kennedy et al., 2010; Akers et al., 2014), further highlighting the need for new anti-biofilm drug therapy.

Several human pathogens have the ability to form biofilms. Thus management of chronic wounds requires compounds effective against multiple antibiotic-tolerant cells housed within biofilms. *Staphylococcus aureus* is a common biofilm-associated pathogen (Chung and Toh, 2014; Percival et al., 2015). In particular, methicillin-resistant *S. aureus* (MRSA) is responsible for one of the leading hospital-acquired infections (Lakhundi and Zhang, 2018). MRSA can cause systemic and topical infections, one of which are chronic wounds (Bhattacharya et al., 2015). In addition to bacteria, yeast-like fungi can cause biofilm-associated infections as well. For example, *Candida* spp. have also been isolated from chronic wound infections, such as diabetic ulcers (Heald et al., 2001) and, similar to *S. aureus*, *Candida* spp. can cause infection both systemically and topically (Mayer et al., 2013). When associated with biofilm, *Candida* spp. are also more tolerant to antifungal agents than in their planktonic form (Cavalheiro and Teixeira, 2018). More recently, *Candida auris* has rapidly become an antifungal resistant pathogen with threatening infection rates (Chowdhary et al., 2017; Jeffery-Smith et al., 2018; Lockhart, 2019). These types of events highlight the need for new lead compounds with the potential to eradicate the planktonic form of a pathogen but also show anti-biofilm potency.

In this study, we investigate the antimicrobial and anti-biofilm properties of Bay 11-7085. This compound was flagged as a hit in our *C. elegans*-MRSA infection high through-put screen (Kim et al., 2018b). In this screen, we tested over 82,000 small molecules to elucidate compounds that inhibit *C. elegans* death after a MRSA infection. This approach has become extremely successful, leading to multiple hit compounds, which have been further studied and characterized for their anti-infective capabilities (Kim et al., 2018a; Kim et al., 2018b; Kim et al., 2020). Due to our *C. elegans*-MRSA whole animal screen system, overlooked compounds, such as those with multiple bioactivities, have now been identified as potent antimicrobials (Escobar et al., 2020). Here we show that our hit compound Bay 11-7085 has antimicrobial and anti-biofilm activity to both *S. aureus* and *Candida* spp. It partially inhibits biofilm initial cell attachment and formation in a polymicrobial co-culture and can partially eradicate mature biofilm. Furthermore, Bay 11-7085 shows activity against *C. albicans* but not *S. aureus* in an *in vitro* multispecies Lubbock chronic wound biofilm model. Bay 11-7085 exhibits a low probability for resistance development to antibiotic pressure against *S. aureus* strain MW2 and can prolong *C. elegans* life from MRSA or *C. albicans* infections.

## Materials and Methods

### Pathogenic Strains and Growth Conditions

All strains used in these studies are included in Supplementary Table S1 *.* Staphylococcal strains were grown overnight in tryptic soy broth (TSB) at 37°C with shaking at 180–225 rpm. Enterococcal strains were grown overnight in brain heart infusion broth (BHI) at 37°C with shaking at 180–225 rpm. All other bacterial strains were grown overnight in Luria-Bertani broth (LB) at 37°C with shaking at 180–225 rpm. *Candida* spp. isolates were grown overnight in Yeast Peptone Dextrose (YPD) at 37°C with shaking at 225 rpm.

### Drugs and Antibiotics

Bay 11-7085 (Tocris 1743), fluconazole, and amphotericin B stocks were dissolved to 10 mg/ml in dimethyl sulfoxide (DMSO). Oxacillin, vancomycin, gentamicin, and ciprofloxacin were dissolved in H_2_O to make 10 mg/ml stocks. Kanamycin was dissolved in H_2_O to make 100 mg/ml stock.

### Minimum Inhibitory Concentration

MIC of bacterial strains were determined by broth microdilution assay according to the Clinical & Laboratory Standards Institute (CLSI) (CLSI, 2012). In brief, strains grown overnight in appropriate media for 20–23 h were diluted to 1 × 10^6^ CFU/ml in Mueller–Hinton Broth (MHB, BD Difco, pH: 7.3 ± 0.1). In a 96-well plate, 50 µl of diluted culture was added to 50 µl of serial two-fold diluted drug in MHB to a final concentration of 5 × 10^5^ CFU/ml. All assays were performed in triplicate. Experimental plates were incubated for 20–22 h at 37°C. Optical density at 600 nm (OD_600_) was quantified using a spectrophotometer (SpectraMax M2, Molecular Devices) as a measure of bacterial growth. MIC was defined as OD_600_ ≤ 0.1 after background subtraction.

The MIC of *Candida* spp. strains were determined by broth microdilution assay according to the CLSI document M27-A using 96-well flat-bottom microtiter plates (Rex, 2008). Briefly, colonies of each strain were inoculated in 5 ml of YPD overnight. The cells were harvested by centrifugation at 4,300 × g for 5 min, washed twice with sterile phosphate-buffered saline (PBS), and adjusted to 1 × 10^3^ cells/ml in RPMI 1640 using a hemocytometer. The compound and antifungal agents used as controls were serially diluted to final concentrations ranging from 0.125 to 64 μg/ml. Candida cells were subsequently added to wells (final concentration 0.5 × 10^3^ cells/ml). Negative controls were performed with RPMI 1640 only, and positive controls with RPMI 1640 and Candida cells only. Microplates were incubated at 37°C without shaking and read at 24 h. The MIC was visually defined as the lowest concentration of compound at which 100% of inhibition was observed, compared with that of the compound-free control. Each assay was performed in triplicate.

### *C. elegans* Infection Assay for Compound Screening

All compounds were screened as previously described in Kim et al. (2018b). In brief, *glp-4* (bn2);*sek-1* (km4) worm embryos were synchronized by plating 2000 L1 worms on slow-kill (SK) agar plates with HB101 bacteria as a food source at 15°C for four days until they reached gravid adult stage. Eggs were harvested and hatched in M9 buffer at 15°C for 48 h. L1 stage worms were then transferred onto SK HB101 plates and incubated at 25°C for 52 h to induce sterility. Sterile young adult stage worms were harvested using M9 buffer and sorted into black, clear-bottom, 384-well plates (Corning no. 3712) containing compounds at 15 worms/well using the Copas Biosort instrument (Union Biometrica, MA, United States). *S. aureus* strain MW2 bacteria were grown overnight in TSB at 37°C with agitation. A static culture was inoculated by seeding 100 µl of overnight culture in 10 ml of fresh TSB, sealed to produce anaerobic conditions, and incubated at 37°C overnight without agitation. We have found that MW2 grown anaerobically elicits a greater infection mortality rate in *C. elegans.* Furthermore, anaerobically grown cells of *S. aureus* strain MW2 express different virulence gene patterns (Fuchs et al., 2007). Static cells of *S. aureus* strain MW2 were added to *C. elegans*-compound 384-well plates at a final concentration of OD_600_ 0.04. The final well composition consisted of 70% M9 buffer, 19% Sheath solution (Union Biometrica Part no. 300-5101-000), 10% TSB, and 1% DMSO or compounds dissolved in DMSO. After a 5-day incubation at 25°C, worms were washed using a multiplate washer and incubated overnight at 37°C with Sytox Orange dissolved in M9 at a final concentration of 0.7 µM. The following day, all plates were imaged using an Image Xpress Micro automated microscope (Molecular Devices, CA, United States), capturing both transmitted light and TRITC (535 nm excitation, 610 nm emission) fluorescent images using a 2× objective.

### *C. elegans* Dose-Dependent Toxicity Assay

In a black, clear-bottom, 96-well plate (Corning, no. 3690), Bay 11-7085 was serially diluted to a final volume of 50 µl using M9. N2 worms were sterilized by growing to young adult stage fed on RNAi *cdc 25.1* activated by 1 mM of IPTG (Yuen and Ausubel, 2018) for 48 h at 25°C. *cdc 25.1* is an integral part of germ cell mitosis. Mutations of *cdc 25.1* inhibit the germ-line cell division producing *C. elegans* incapable of laying eggs (Kim et al., 2009). Young adult worms were washed 3 times with 50 ml of M9 and diluted to an average of 21 worms ± 7 per 25 µl using a multichannel pipette. An additional 25 µl of heat-killed OP50 were added to each well to make a final volume of 100 µl and OD_600_ of 0.5. All assays were performed in duplicate. Experimental plates were incubated for 24 h at 25°C. Worms were then washed using a 405 LS microplate washer (BioTek, VT, United States) and incubated with 0.7 µM Sytox Orange for an additional 24 h at 25°C. Each plate was then imaged using an Xpress Micro automated microscope (Molecular Devices, CA, United States), capturing both transmitted light and TRITC (535 nm excitation, 610 nm emission) fluorescent images using a 2× objective. Surviving worms were considered those with no TRITC signal relative to the control.

### Bacterial and Fungal Time-Course Killing Assay

Overnight culture *S. aureus* strain MW2 was diluted 1:100 in TSB and grown to log phase (OD_600_ 0.3–0.6). The log phase culture was then diluted 1:100 (2 × 10^6^ CFU/ml) in MHB (BD Difco, pH 7.3 ± 0.1). 250 µl of diluted culture was added to 250 µl of serial two-fold diluted drug in MHB to a final concentration of 1 × 10^6^ CFU/ml. Plates were incubated at 37°C with agitation (225 rpm). At time-points 0, 1, 2, 3, 4, and 24 h, 50-μl samples were removed, diluted 5-fold to sub-MIC levels, then serially diluted by 10-fold steps, and lastly spot-plated on TSB agar (BD Difco) plates to enumerate CFU/ml. These experiments were conducted in triplicate. Experimental plates were incubated for 20–22 h at 37°C.

Log-phase cultures of *C. albicans* were regrown and adjusted to a density of 10^6^ cells/ml in RPMI 1640 and added to growth medium alone or specific concentrations of the compound being tested. Plates were incubated at 37°C with agitation (225 rpm). At predetermined time points (0, 6, 12, 24, 48 h), 50 μl aliquots were obtained from each solution, serially diluted in YPD broth, and 5 μl were plated on YPD agar plates for 24 h at 37°C for determination of CFU/ml. Three independent experiments were performed in triplicate.

### Human Blood Hemolysis

Hemolytic activity of Bay 11-7085 on human erythrocytes was evaluated using a previously described method with modifications (Rajamuthiah et al., 2015). 10% human erythrocytes were purchased from Rockland Immunochemicals (Limerick, PA, United States). The erythrocytes were diluted to 4% in PBS, and 50 μl were added to 50 μl of two-fold serial dilutions of compound in PBS, 0.2% DMSO (negative control), or 1% Triton-X 100 (positive control) in a 96-well plate. Plates were incubated at 37°C for 1 h and then centrifuged at 500×*g* for 5 min. 50 μl of the supernatant was transferred to a fresh 96-well plate, and absorbance (A) was measured at 540 nm. Percent hemolysis was calculated using the following equation: (A540 nm of compound treated sample—A540 nm of 0.1% DMSO treated sample)/(A540 nm of 1% Triton X-100 treated sample—A540 nm of 0.1% DMSO treated sample) × 100. All experiments were conducted in triplicate.

### Mammalian Cell Viability Assay

Human hepatoma cell line HepG2 cells were grown in DMEM 10% FBS to confluency and seeded onto 96-well drug plates at 1 × 10^6^ cells/ml. Human renal proximal tubule epithelial RPTEC cells were grown in Renal Epithelial Cell Basal Medium (ATCC PCS-400-030) with supplements (Renal Epithelial Cell Growth Kit ATCC PCS-400-040) and seeded onto 96-well drug plates at 1 × 10^6^ cells/ml. Two-fold concentration drug plates were prepared using corresponding growth media for each cell. Drug and cell plate were then incubated for 22 h at 37°C and 5% CO_2_. At 22 h, 10 μl of WST-1 (Roche, Sigma) was added to each well, following manufacturer’s directions, and incubated for an additional 2 h. Plate absorbance was read at 450 nm. Samples were normalized to a non-treatment control. All experiments were conducted in triplicate.

### Biofilm Initial Cell Attachment Assay

#### Monoculture

Absorbance (OD_600_) of overnight culture *S. aureus* strain VRS1 was measured and adjusted to 0.2 in BHI + 0.1% glucose medium. 100 µl of bacteria was added to 2× drug concentrations being tested (diluted in BHI +0.1% glucose), producing a final volume of 200 μl, OD_600_ of 0.1 (∼1 × 10^8^ CFU/ml) and 1 × drug concentration. The plates were then incubated at 37°C for 1 h. After incubation, the culture was pipetted out, and the wells washed 3 times with PBS to remove any non-adherent planktonic cells. Biofilm initial cell attachment was measured as described in Biswajit et al., 2016 (Mishra et al., 2016). In brief, colorimetric quantification of the inhibition of biofilm initial cell attachment was done using XTT [2–3-bis(2-methyloxy-4-nitro-5-sulfophenyl)-2H-tertazolium-5-carboxanilide] assay kit following manufacture instructions with minor adjustments (Sigma-Aldrich, MO, United States). 180 µl of fresh BHI and 20 µl of XTT working solution were added to each well, and the plates were again incubated for 2 h at 37°C. Absorbance at 450 nm was measured, and each experimental well was normalized to a non-treatment control. Each biological replicate was done in quadruplicates. One replicate was done in octuplet. Three total biological replicates were conducted in total.

For a *Candida* spp. biofilm assay, 50 µl of RPMI 1640 media containing Bay 11-7085 was serially diluted in a 96-well polystyrene microtiter plate. Overnight culture of either *Candida* spp. was washed twice with PBS, resuspended in RPMI 1640 (final concentration: 1 × 10^7^ cells/ml), and added to each well to a final volume of 100 µl. Each plate was then statically incubated for 90 min at 37°C. Following incubation, plates were washed twice with PBS, and cell attachment was measured using XTT assay kit following manufactures instructions with minor modification (Khan et al., 2017). In brief, a working solution of XTT was prepared where 80 μl of PBS and 20 μl of XTT were added to each well. Plates were then incubated in the dark for 2 h at 37°C, and absorbance was measured at 490 nm using a microtiter plate reader. Experimental wells were normalized to a non-treatment control and background subtraction. Three biological replicates were conducted in octuplet.

#### Co-culture

Co-cultured *S. aureus* and *C. albicans* was executed as previously described (Harriott and Noverr, 2009). In detail, *S. aureus* strain VRS1 (Weigel et al., 2003) and *C. albicans* strain SC5314 were grown overnight with agitation in BHI or YPD respectively at 37°C, shaking at 225 rpm. Cells were then washed twice with sterile PBS. VRS1 was adjusted to OD_600_ 0.4 (∼10^7^ CFU/ml), and *C. albicans* was adjusted to 2 × 10^6^ CFU/ml by counting cells using a hemocytometer. Washed cells were then diluted in 50% serum-BHI medium at a 1:10 ration *S. aureus* to *C. albicans*. Drug plates were prepared using 50% serum-BHI medium and 2× concentration of each drug dose tested in a 24-well plate. A Millipore mixed cellulose ester membrane disk (GSWP01300, EMD Millipore, Billerica, MA, United States) was placed in each well containing 500 µl of 2× drug concentration. 500 µl of co-culture mix was added to each well of the drug plate, making a final volume of 1 ml, 10^5^–10^7^ CFU/ml *S. aureus*, and 10^6^ *C. albicans.* Disks were then incubated at 37°C for 90 min, washed 3 times with PBS, and transferred to a 1.5 ml microtube containing equal amounts of PBS. 200 µl were aliquoted in a fresh microtube and stored for later. Microtubes containing disks were sonicated for 10 min using an ultrasonic bath (Fisher Scientific FS 30). Both pre-sonicated and post-sonicated samples were then collected from each tube and serially diluted on selective plates to enumerate total CFU/membrane of *S. aureus* and *C. albicans.* Selective plates used were mannitol salt (BD Difco, pH: 7.4 ± 0.2) incubated at 37°C (for *S. aureus* strain VRS1) and YPD (Research Products International) containing 100 μg/ml of kanamycin incubated at 25°C (for *C. albicans* strain SC5314). Each sample was normalized to pre-sonicated samples. Three biological replicates were conducted in duplicate.

### Biofilm Inhibition and Eradication Assay

#### Monoculture

Absorbance (OD_600_) of overnight culture *S. aureus* strain VRS1 was measured and adjusted to 0.1 in BHI + 0.1% glucose medium. 100 µl of bacteria was added to 2× drug concentrations (diluted in BHI + 0.1% glucose medium) producing a final volume of 200 µl (∼1 × 10^7^ CFU/ml) and 1× drug concentration. After 24 h of static incubation at 37°C, media was pipetted out, and wells were washed 3 times with PBS to remove any non-adherent planktonic cells. Biofilm formation was measured using crystal violet (CV) (O'Toole, 2011). In brief, plates were incubated with 1% CV for 15 min at room temperature. Plates were then washed 3 times with PBS and dissolved with 200 µl of 30% acetic acid. Absorbance at 550 nm was measured and each experimental well was normalized to a non-treatment control. Three biological replicates were conducted in octuplet.

For biofilm eradication, biofilms were prepared as described above, omitting the initial drug dose. After static incubation at 37°C for 24 h, plates were washed 2 times with PBS. Various concentrations of drug tested were diluted in BHI + 0.1% glucose medium and added to each well to a final volume of 200 µl. After an additional incubation of 24 h plates were washed 3 times with PBS and stained with CV as described previously. Three biological replicate were conducted in octuplet.

For *Candida* spp., overnight suspensions were centrifuged (4,000×g for 5 min), washed twice with PBS, and resuspended in RPMI 1640. 50 µl of RPMI 1640 containing Bay 11-7085 were serially diluted in a 96-well polystyrene microtiter plate. 50 μl aliquots of fungal suspensions (final concentration: 1 × 10^6^ cells/mL) were transferred to each well of the microtiter plate and incubated statically for 24 h at 37°C. Unseeded wells (with RPMI 1640 only) acted as the negative background control for the subsequent steps. Following incubation, the biofilm was assessed using XTT (Mishra et al., 2016), as described in the section: *Biofilm Initial Cell Attachment Assay*, or CV (O'Toole, 2011). For CV methodology, cells were fixed with methanol for 15 min and stained with 100 μl of 0.4% CV solution for 15 min. Any excess CV was removed by washing with sterile water before adding 100 μl of absolute ethanol to release the dye from the biofilm. After 30 min, the absorbance was measured at 570 nm using a microtiter plate reader. Three biological replicate were conducted in octuplet.

For biofilm eradication, biofilms were prepared as described above, omitting the initial drug dose. After static incubating at 37°C for 24 h, plates were washed 2 times with PBS, and various drug tested concentrations were added to each well to a final volume of 100 µl. After an additional incubation of 24 h, plates were washed 3 times with PBS and assessed using XTT (Mishra et al., 2016) or stained with CV (O'Toole, 2011) as described above. Three biological replicate were conducted in octuplet.

#### Co-culture

Co-cultured *S. aureus* and *C. albicans* were executed as previously described (Harriott and Noverr, 2009). In detail, *S. aureus* strain VRS1 (Weigel et al., 2003) and *C. albicans* strain SC5314 were grown overnight with agitation in BHI or YPD, respectively, at 37°C. Cells were then washed twice with sterile PBS. VRS1 was adjusted to OD_600_ 0.4 (∼10^7^ CFU/ml), and *C. albicans* was adjusted to 2 × 10^6^ CFU/ml in PBS by counting cells using a hemocytometer. Washed cells were then diluted in 50% serum-BHI medium (serum: Fetal bovine serum, ThermoFisher, Remel, Waltham, MA) at a 1:10 ration *S. aureus* to *C. albicans*. Drug plates were prepared by serially diluting 100 µl of 50% serum-BHI medium and 2× concentration of each drug dose tested in a 96-well plate. 100 µl of co-culture mix was added to each well of the drug plate, making a final volume of 200 μl, 10^5^–10^7^ CFU/ml VRS1, and 10^6^ *C. albicans.* After 24 h of static incubation at 37°C, media was pipetted out, and wells were washed 3 times with PBS to remove any non-adherent planktonic cells. Biofilm formation was measured using CV (O'Toole, 2011). In brief, plates were incubated with 1% CV for 15 min at room temperature. Plates were then washed 3 times with PBS and dissolved with 200 µl of 30% acetic acid. Absorbance at 550 nm was measured and each experimental well was normalized to a non-treatment control. Each biological replicate was done in octuplet.

For biofilm eradication, biofilms were prepared as described above, omitting the initial drug dose. After static incubating at 37°C for 24 h, plates were washed 2 times with PBS. Various drug concentrations, diluted in BHI + 0.1% glucose medium, were added to each well to a final volume of 200 µl. After an additional incubation of 24 h plates were washed 3 times with PBS and stained with CV as described previously.

### *In vitro* Multispecies Lubbock Chronic Wound Biofilm Model

*S. aureus* strain VRS1 and *C. albicans* strain SC5314 were co-cultured using the *in vitro* multispecies Lubbock chronic wound biofilm model (Sun et al., 2008). Wound like media (WLM) was composed of 50% Bolton Broth (Sigma Aldrich, Oxoid, St. Louis, MO), 45% heparinized calf serum (Rockland, Limerick, PA), and 5% laked horse blood (Thermofisher, Remel, Waltham, MA). In detail, *S. aureus* strain VRS1 (Weigel et al., 2003) and *C. albicans* strain SC5314 were grown overnight with agitation in BHI or YPD, respectively at 37°C. Cells were than washed twice with sterile PBS and diluted into WLM. *S. aureus* was adjusted to OD_600_ 0.04 (∼10^6^ CFU/ml), and *C. albicans* was adjusted to 1 × 10^7^ CFU/ml using a hemocytometer. Drug plates were prepared by serially diluting 50 µl WLM and 2× concentration of each drug dose tested in a 96-well plate. 50 µl of VRS1-*C. albicans* co-culture mix was added to each well of the drug plate, making a final volume of 100 μl, 10^5^–10^6^ CFU/ml VRS1, and 10^7^ CFU/ml *C albicans. S. aureus* strain VRS1 is coagulase-positive and therefore created a jelly-like substance that served as a scaffold for biofilm formation (DeLeon et al., 2014). After 24 h of static incubation at 37°C the complete coagulated jelly-mass from each well was transferred to individual 1.5 ml microtubes containing 1 ml of sterile PBS. Tubes were vortexed at the highest setting for 2 min and then sonicated for 10 min to release cells from the jelly mass. 100 µl of each tube was 10-fold serially diluted and spot platted on selective plates in order to enumerate total CFU/ml of *S. aureus* and *C. albicans.* Selective plates used were mannitol salt (BD Difco, pH: 7.4 ± 0.2) incubated at 37°C (*S. aureus*) and YPD (Research Products International) containing 100 μg/ml of kanamycin incubated at 25°C (*C. albicans*). All experiments were conducted in triplicate.

### *C. elegans*-*Candida* spp. Infection Assay

*C. elegans* strain *glp-4;sek-1* (AU37) was maintained on *Escherichia coli* OP50 spread on nematode growth medium agar plates and incubated at 15°C. For *C. elegans-Candida* killing assay, worms were synchronized and grown to young adult stage by incubating 24 h at 15°C and 72 h at 25°C. Worms were collected and washed 3 times with PBS and transferred to a *C. albicans* lawn grown on a BHI plate overnight at 37°C. Worms on *C. albicans*/BHI plates were incubated at 25°C for 4 h and then washed 4 times with PBS. Thirty to seventy five worms were dispensed into each well of a 6-well plate containing desired drug concentration diluted in 20% BHI and 45 μg/ml kanamycin to a total volume of 2 ml. Worms were incubated at 25°C and scored daily for dead worms for a total of 6 days. Statistical significance was displayed using Kaplan-Meier survival analysis.

## Results

### Bay 11-7085 Shows Anti-Staphylococcal Activity *in vitro* and a Whole Animal *C. elegans* Infection Model

**FIGURE 1:**
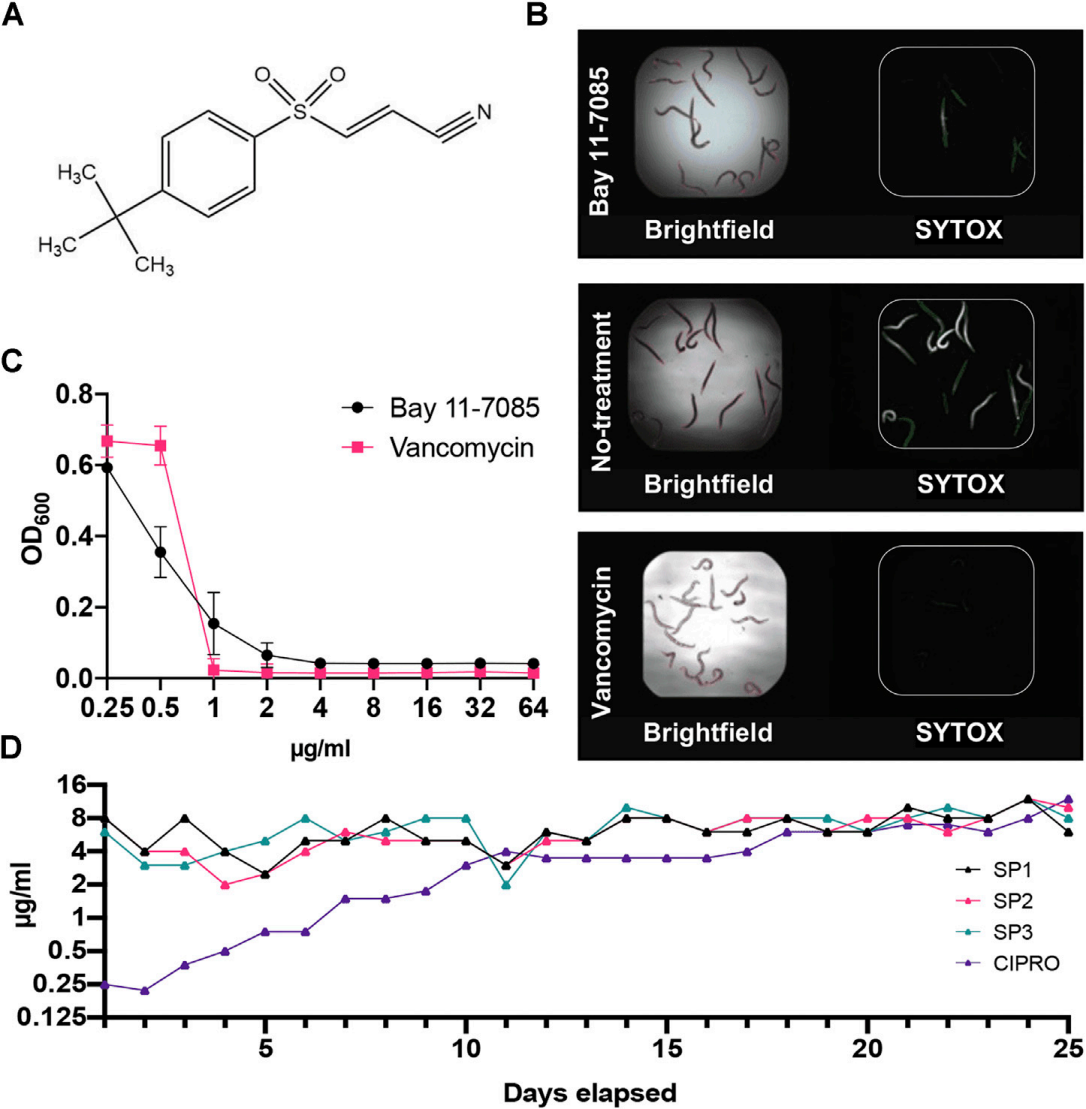
Bay 11-7085 rescues *C. elegans* from a MRSA infection and shows low antibiotic resistance to *S. aureus* strain MW2. **(A)** Chemical structure of Bay 11-7085. **(B)** Fifteen MRSA-infected *C. elegans* were treated with 7.14 μg/ml Bay 11-7085, 0.1% dimethyl sulfoxide (DMSO) (no-treatment) or 10 μg/ml vancomycin for 5 days. After staining dead worms with Sytox Orange, brightfield **(left)** and fluorescence **(right)** images were obtained. **(C)** *In vitro* activity of Bay 11-7085 and antibiotic control vancomycin against MRSA strain MW2. Bacterial growth is quantified as a measure of OD_600_. **(D)** Three individual MRSA strain MW2 replicates (SP1-3) were serially passaged in increasing concentrations of Bay 11-7085 for 25 days. Passages with ciprofloxacin (CIPRO) were conducted in parallel as a control.

Through our *C. elegans*-MRSA infection model, we identified Bay 11-7085 (Figure 1A) as a hit compound able to save *C. elegans* from a lethal MRSA infection. Our screen showed that Bay 11-7085 saved 12 out of the 15 infected worms at a concentration of 7.14 μg/ml (Figure 1B). Relative to vancomycin, a common Gram-positive antibiotic, Bay 11-7085 showed a distinct antimicrobial profile (Figure 1C) with a MIC of 4 μg/ml (Table 1).

**TABLE 1:** Minimum inhibitory concentrations (µg/ml) of Bay 11-7085 and conventional antibiotics against various pathogenic strains.

Strain	Bay 11-7085	Oxa	Van	Gm	Cipro	FLC
*Staphylococcus aureus* MW2	4	64	1	0.5	0.25	n.d.
*Staphylococcus aureus* LAC	2	8	0.5	0.25	8	n.d.
*Staphylococcus aureus* WKZ-1	4	0.5	1	0.5	1	n.d.
*Staphylococcus aureus* WKZ-2	2	64	1	0.5	1	n.d.
*Staphylococcus aureus* ATCC 25923	2	0.25	2	0.25	0.25	n.d.
*Staphylococcus aureus* ATCC 29213	2	0.25	1	0.5	0.5	n.d.
*Staphylococcus aureus* ATCC 43300	4	16	1	>64	0.5	n.d.
*Staphylococcus aureus* VRS1	4	>64	>64	64	64	n.d.
*Enterococcus faecium* E007	64	16	4	>64	1	n.d.
*Enterococcus faecalis* MMH 594	64	32	2	>64	0.5	n.d.
*Klebsiella pneumoniae* KCTC2242	>64	>64	>64	1	<0.0625	n.d.
*Klebsiella pneumoniae* WGLW2	>64	>64	>64	1	<0.0625	n.d.
*Pseudomonas aeruginosa* ATCC 27853	>64	>64	>64	4	0.5	n.d.
*Pseudomonas aeruginosa* PA14	>64	>64	>64	2	<0.0625	n.d.
*Enterobacter aerogenes* KCTC 2190	>64	>64	>64	1	<0.0625	n.d.
*Candida albicans* SC5314	1	n.d.	n.d.	n.d.	n.d.	0.25
*Candida glabrata* ATCC 90030	1	n.d.	n.d.	n.d.	n.d.	8
*Candida parapsilosis* ATCC 22019	8	n.d.	n.d.	n.d.	n.d.	1
*Candida auris* AR-BANK #0381 (CDC)	0.5	n.d.	n.d.	n.d.	n.d.	n.d.

Oxa, oxacillin; Van, vancomycin; Gm, gentamicin; Cipro, ciprofloxacin; FLC, fluconazole.

To assess the rate of antimicrobial resistance to Bay 11-7085, we conducted serial passage resistance development by treating *S. aureus* strain MW2 with sub-MIC levels of Bay 11-7085 for 25 days. After 25 serial passages, *S. aureus* strain MW2 was unable to acquire resistance (Figure 1D). However, did gain resistance to ciprofloxacin up to 32× its original MIC of 0.25 μg/ml (Figure 1D). These results indicate that Bay 11-7085 has a low probability of antibiotic resistance over an extended period of time.

### Bay 11-7085 Shows Antimicrobial activity to *S. aureus* and Fungal *Candida* spp.

**FIGURE 2:**
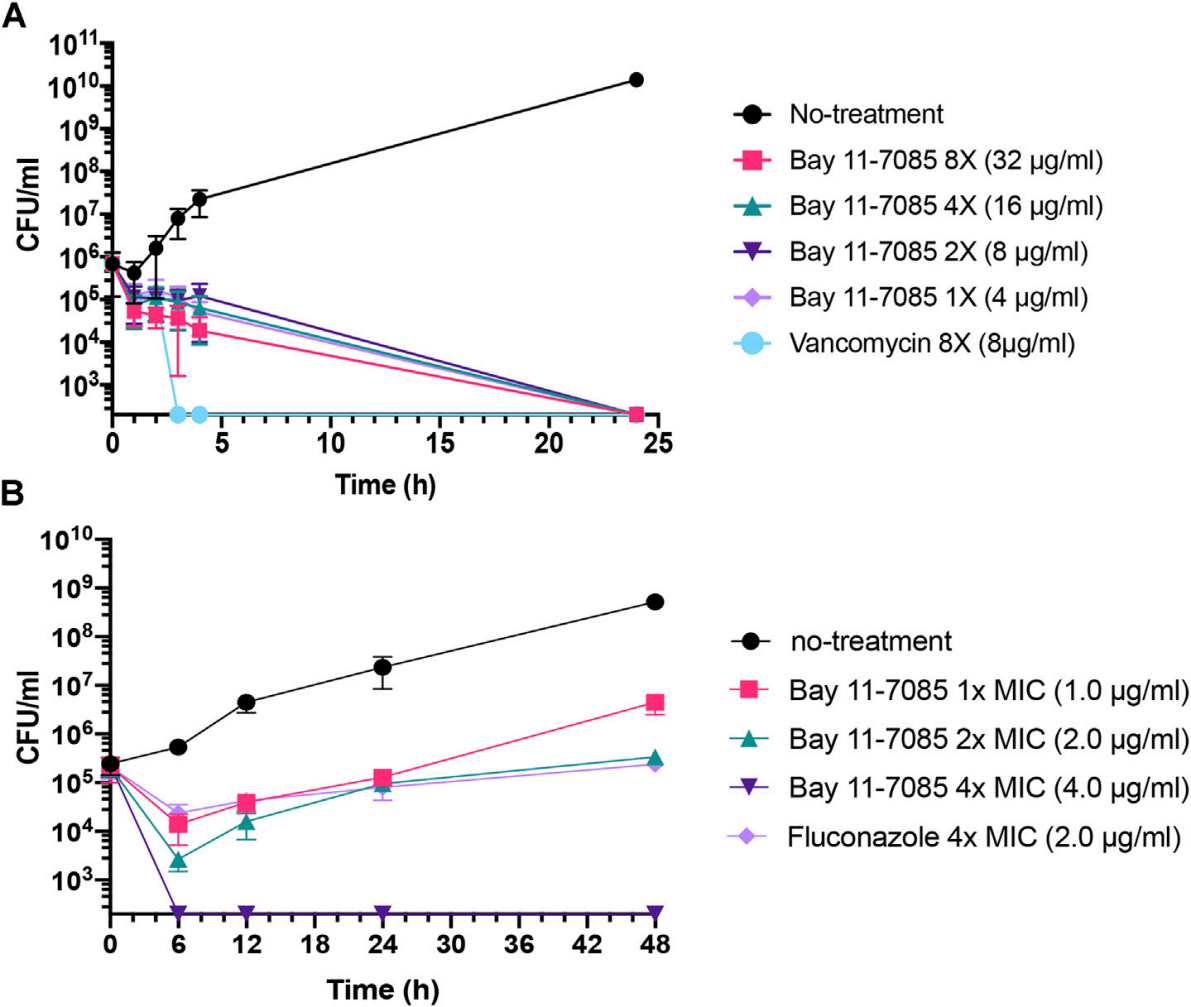
Bay 11-7085 is bactericidal toward MRSA and fungistatic toward *C. albicans*. **(A)** MRSA strain MW2 cells were grown to log phase OD_600_ 0.3–0.4 from overnight liquid culture. Log phase cells were diluted to 10^6^ CFU/ml in various concentration of Bay 11-7085 and 8 μg/ml of vancomycin as a positive control. **(B)** Overnight culture of *C. albicans* strain SC5314 was diluted to ∼10^6^ CFU/ml and added to 1× (1 μg/ml), 2× (2 μg/ml) and 4× (4 μg/ml) MIC of Bay 11-7085. Fluconazole was used as a positive control at 4× MIC (2.0 μg/ml). Samples were collected at various time points, serially diluted, and plated to measure total CFU/mL. (*n* = 3, ±S.D.).

To further test Bay 11-7085s antimicrobial capability, we tested its activity against a panel of ESKAPE pathogens. The ESKAPE pathogens include two Gram-positive bacteria (*Enterococcus* spp. and *S. aureus*) and four Gram-negative bacteria (*Klebsiella pneumoniae*, *Acinetobacter baumannii*, *Pseudomonas aeruginosa*, and *Enterobacter* spp.) (Pendleton et al., 2013; Rice, 2008). Bay 11-7085 did not show activity toward any Gram-negative species of bacteria with a MIC ≥ 64 μg/ml (Table 1) and, unlike *S. aureus* strain MW2, we observed nominal activity toward other Gram-positive pathogens such as *E. faecalis* or *E. faecium* with a MIC of 64 μg/ml for both species (Table 1). To further support these findings, we tested a panel of *S. aureus* strains, including vancomycin-resistant *S. aureus* strain VRS1 (Weigel et al., 2003), and found that indeed Bay 11-7085 had a MIC of 2–4 μg/ml against various *S. aureus* strains (Table 1). Interestingly, when we tested Bay 11-7085 bactericidal or bacteriostatic effect, our time-course killing data show that Bay 11-7085 is bactericidal to log phase *S. aureus* strain MW2 cells at MIC and higher (Figure 2A). Taken together, these findings suggest that Bay 11-7085 is not a broad bactericidal antibiotic but instead shows specific potency toward *S. aureus* strains.

Next, we questioned if Bay 11-7085 has antifungal activity. As detailed above*, Candida* spp. can cause chronic infection both systemically and topically. To answer this, we measured the MIC of Bay 11-7085 to four *Candida* spp.: *C. albicans*, *Candida glabrata*, *Candida parapsilosis*, and *C. auris* after 24 h of treatment. Strikingly, we found that Bay 11-7085 had a low MIC of 0.5–1 μg/ml for 3 of the 4 species tested (Table 1), while the MIC to *C. parapsilosis* was 8 μg/ml (Table 1). Of particular interest is the activity observed toward *C. auris*. To confirm the activity of Bay 11-7085 toward *C. auris* we expanded our MIC profiling to 10 total *C. auris* antifungal resistant strains acquired from the Center for Diseases Control and Prevention (CDC) clinical isolate bank. Indeed, we found that Bay 11-7085 had a low MIC of 0.5–1 μg/ml to multiple antifungal resistant *C. auris* clinical strains (Supplementary Table S2). Given that Bay 11-7085 is bactericidal to MRSA strain MW2, we hypothesized that Bay 11-7085 would work similarly toward *Candida* spp. To evaluate our hypothesis, we performed a time-kill kinetics assay using *C. albicans* strain SC5314. In contrast to *S. aureus* (Figure 2A), Bay 11-7085 is unable to completely kill *C. albicans* (Figure 2B) at 1–2× MIC. However, at a higher concentration such as 4× MIC (4 μg/ml), Bay 11-7085 is entirely fungicidal. Taken in their totality, we conclude that Bay 11-7085 can inhibit fungal growth at a low concentration such as 1–2 μg/ml but completely fungicidal at a higher concentration such as 4 μg/ml.

### Cytotoxicity of Bay 11-7085 on Human Cells and *C. elegans*

**FIGURE 3:**
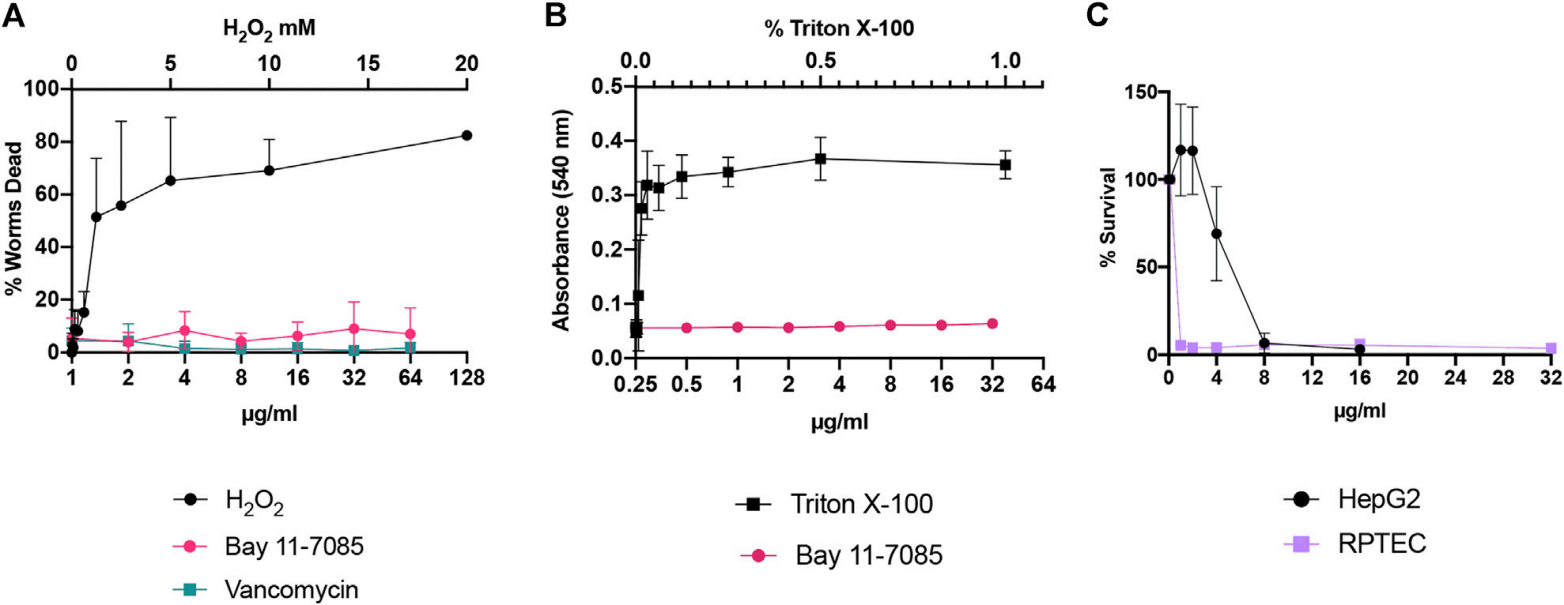
Bay 11-7085 shows varied toxicity to *C. elegans*, human red blood cells, and mammalian cells. Bay 11-7085s toxicity profile to **(A)** *C. elegans*, **(B)** human red blood cells, and **(C)** mammalian cells at multiple concentrations. X-axis represents concentrations of Bay 11-7085 in µg/ml. Hydrogen peroxide (H_2_O_2_) was used as a positive control in our *C. elegans* toxicity assay. Triton-X 100 was used as a positive control in our human red blood cell lysis assay. (*n* = 3, ±S.D.).

Next, we assessed the toxicity profile of Bay 11-7085 *in vitro* and *in vivo*. *C. elegans* are widely used as a model organism for toxicology studies (Uring-Lambert et al., 1987; Hunt et al., 2018; Wittkowski et al., 2019). Therefore, we first tested toxicity to *C. elegans* at various concentration of Bay 11-7085 from 1 μg/ml up to 64 μg/ml (Figure 3A). Our data support that Bay 11-7085 is minimally toxic to *C. elegans* with greater than 90% survival after 24 h of exposure (Figure 3A). When we subjected human red blood cells to Bay 11-7085, we found no indication of hemolysis at a concentration up to 32 μg/ml (Figure 3B). However, when we examined Bay 11-7085s toxicity to human hepatoma cell line HepG2 and primary human renal proximal tubule epithelial cells (RPTEC), we recorded an IC_50_ of 4.6 μg/ml and 0.96 μg/ml (Figure 3C), which is nearly equal to or lower than Bay 11-7085s MIC to *S. aureus* strain MW2 (4 μg/ml). Given the toxicity of Bay 11-7085 to primary cells, we conclude that Bay 11-7085 has the most potential for topical application, such as wound management.

### Bay 11-7085 has Anti-Biofilm Activity Toward Three Phases of *S. aureus* Biofilm Maturation

**FIGURE 4:**
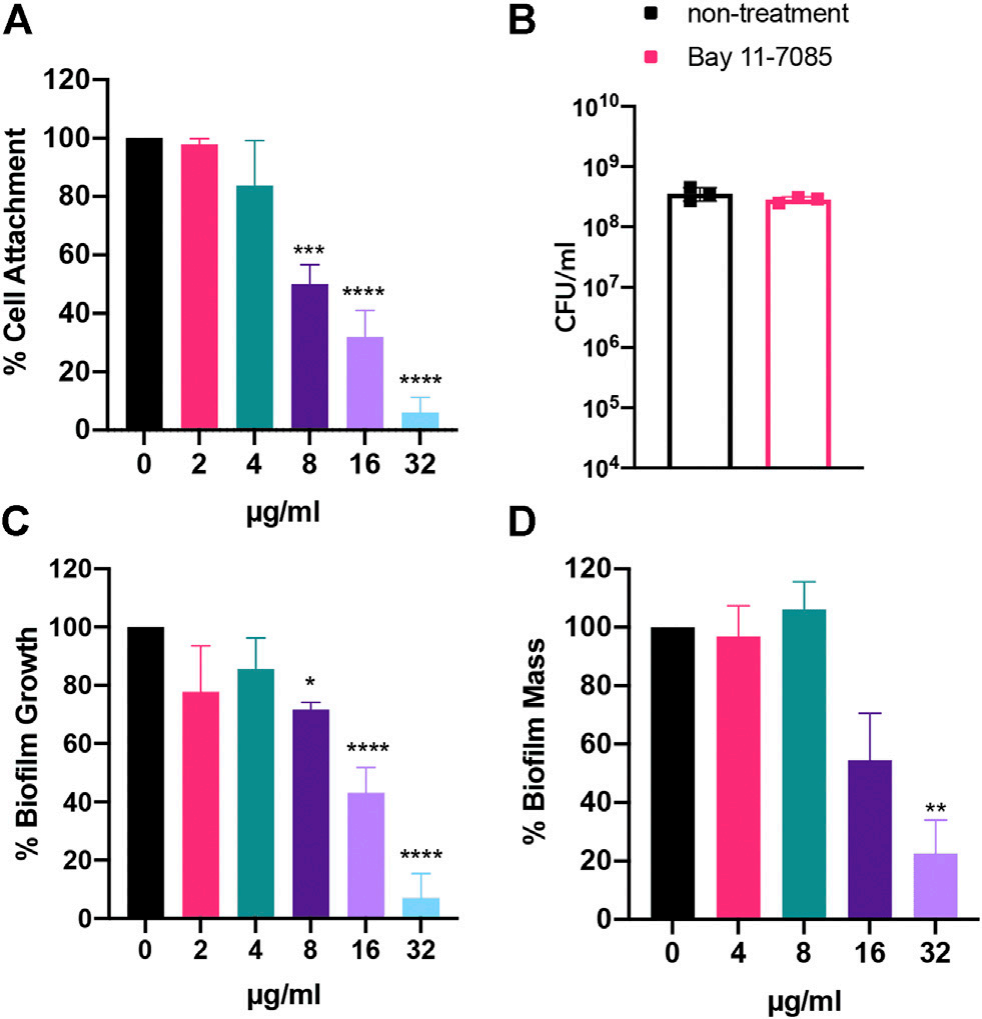
Bay 11-7085 shows anti-biofilm activity toward three phases of *S. aureus* strain VRS1 biofilm maturation. Efficacity of Bay 11-7085 at various concentrations were tested against *S. aureus* strain VRS1 biofilm **(A)** initial cell attachment. **(B)** CFU/ml was measured of treated and untreated conditions after initial cell attachment assay. *S. aureus* strain VRS1 biofilm **(C)** inhibition and **(D)** eradication was measured using 1% crystal violet. X-axis represents concentrations of Bay 11-7085 in µg/ml. Statistical significance was determined using one-way ANOVA with Dunnett’s test for multiple comparisons. *p < 0.05; **p < 0.01; ***p < 0.001; ****p < 0.0001. (*n* = 3, ±S.D.).

Given Bay 11-7085s antimicrobial effect on *S. aureus* strains, we wondered if Bay 11-7085 would have any effect on the stages of *S. aureus* biofilm maturation cycle: initial cell attachment, formation, and mature biofilm. To test this, we first measured if Bay 11-7085 had any activity on the first stage of *S. aureus* biofilm formation, initial cell attachment (Moormeier and Bayles, 2017). To begin, we first tested the potency of various antibiotic-resistant *S. aureus* strains to form biofilm. Our trials revealed that *S. aureus* strain VRS1 (Weigel et al., 2003), a vancomycin-resistant strain, was able to form a more robust biofilm than other antibiotic-resistant *S. aureus* strains such as MW2 (data not shown). We, therefore, decided to move forward with VRS1 in our biofilm studies. Subsequently, we found that Bay 11-7085 was able to inhibit initial cell attachment by >60% beginning at 8 μg/ml (2× MIC) and as much as >90% at 32 μg/ml (8× MIC) (Figure 4A). Additionally, we also confirmed that this activity was not due to the direct killing of *S. aureus* by Bay 11-7085 (Figure 4B) by measuring total CFU/ml (colony-forming units per ml) before and after treatment.

From our previous results, we then hypothesized that Bay 11-7085s activity on initial cell attachment would lead to complete or partial inhibition of *S. aureus* strain VRS1 biofilm formation. We found that Bay 11-7085 can significantly begin to inhibit *S. aureus* biofilm formation at concentrations as low as 8 μg/ml with >20% inhibition (Figure 4C). However, at high concentrations such as 32 μg/ml Bay 11-7085 had an activity of >90% inhibition relative to no-treatment control (Figure 4C). Lastly, we wanted to test if Bay 11-7085 would affect fully mature biofilm of *S. aureus* strain VRS1. Indeed, at a higher concentration of 32 μg/ml Bay 11-7085 was able to eradicate biofilm >70% (Figure 4D). Given these data we conclude that Bay 11-7085 is a potent anti-biofilm compound with activity on initial cell attachment, biofilm formation, and eradicating mature biofilm.

### Bay 11-7085 has Anti-Biofilm Activity Toward Three Phases of *C. albicans* and *C. auris* Biofilm Maturation

**FIGURE 5:**
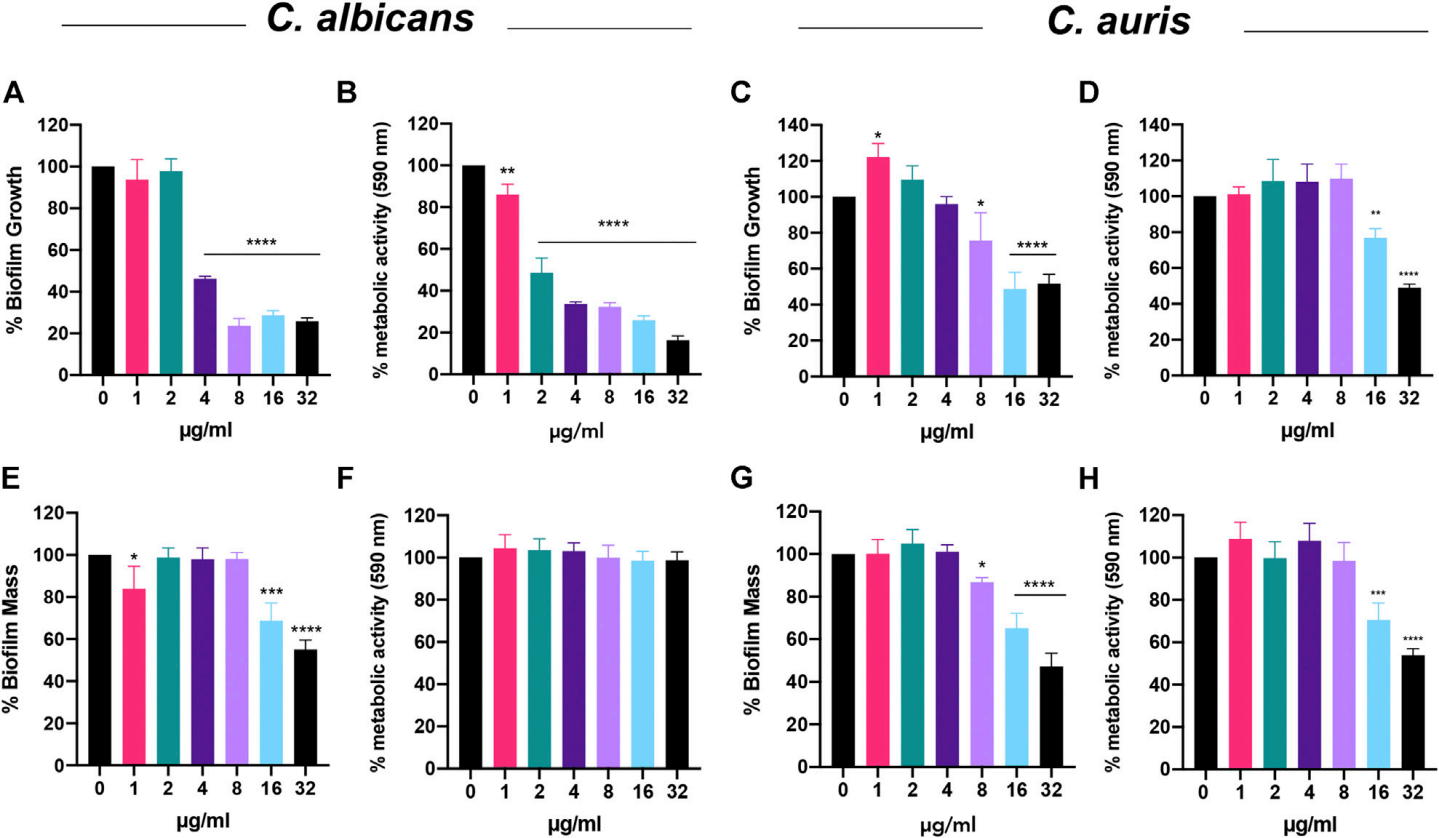
Bay 11-7085 shows anti-biofilm activity toward three phases of *C. albicans* and *C. auris* biofilm maturation. Efficacity of Bay 11-7085 was measured against *C. albicans* strain SC5314 and *C. auris* strain #0384 biofilm inhibition and eradication. Total biomass was measured using 1% crystal violet (CV). Metabolic activity was measured using XTT (590 nm). Inhibition of *C. albicans* biofilm formation by various concentrations of Bay 11-7085 as measured by **(A)** 1% CV and **(B)** XTT dye. Inhibition of *C. auris* biofilm formation by various concentrations of Bay 11-7085 as measured by **(C)** 1% CV and **(D)** XTT dye. *C. albicans* biofilm eradication as measured by **(E)** 1% CV, and **(G)** XTT dye. *C. auris* biofilm eradication as measured by **(G)** 1% CV and **(H)** XTT dye. X-axis represents concentrations of Bay 11-7085 in µg/ml. Statistical significance was determined using one-way ANOVA with Dunnett’s test for multiple comparisons. *p < 0.05; **p < 0.01; ***p < 0.001; ****p < 0.0001. (*n* = 3, ±S.D.).

Next, we questioned whether Bay 11-7085 would also show potency on fungal biofilms. To answer this, we first tested if Bay 11-7085 would inhibit *C. albicans* strain SC5314 and *C. auris* strain #0384 biofilm formation. For our studies, we decided to measure total biofilm mass (including total ECM) using crystal violet (CV) methodology as well as total metabolically active cells using colorimetric dye XTT [2–3-bis(2-methyloxy-4-nitro-5- sulfophenyl)-2H-tertazolium-5-carboxanilide]. After 24 h of incubation, we found that Bay 11-7085 can inhibit maturation of *C. albicans* total biofilm mass by >50% starting at 4 μg/ml (4× MIC) and as much as >70% at 8–32 μg/ml (8–32× MIC) (Figure 5A). In addition, *C. albicans* biofilm-forming cell viability was significantly decreased >15% starting at 1 μg/ml (1× MIC) and up to >50%, >60%, and >80% at 2, 4–16, and 32 μg/ml, respectively (Figure 5B). After testing *C. auris* we found that compound Bay 11-7085 inhibited total biofilm mass maturation starting at 8 μg/ml with >20% inhibition and as much as >60% at 16–32 μg/ml (Figure 5C). Furthermore, we found that Bay 11-7085 decreased cell metabolic activity by >20% at 16 μg/ml and 45% at 32 μg/ml (Figure 5D). From this data, we conclude that Bay 11-7085 can successfully inhibit the maturation of total biofilm mass, including ECM, comparable to total cell growth.

When we tested the most difficult to treat mature biofilm, we found that Bay 11-7085 began to eradicate *C. albicans* total biofilm mass beginning at 16 μg/ml with >30% eradication and >40% eradication at 32 μg/ml (Figure 5E). Surprisingly, after treating mature biofilm with various concentrations of Bay 11-7085 and subsequently incubating with XTT dye we found that Bay 11-7085 is ineffective at decreasing cell viability within *C. albicans* mature biofilm (Figure 5F). Moreover, *C. auris* biofilm mass was significantly eradicated beginning at 8 μg/ml at >10% and >30% at 16 μg/ml and >50% at 32 μg/ml, respectively (Figure 5G), while metabolic activity was not changed at 8 μg/ml but significantly decreased >30% at 16 μg/ml and >45% at 32 μg/ml (Figure 5H). We thus conclude that Bay 11-7085 is effective at decreasing the ECM of mature *Candida* spp. biofilm but not potent enough to completely kill biofilm residing cells of both species tested.

**FIGURE 6:**
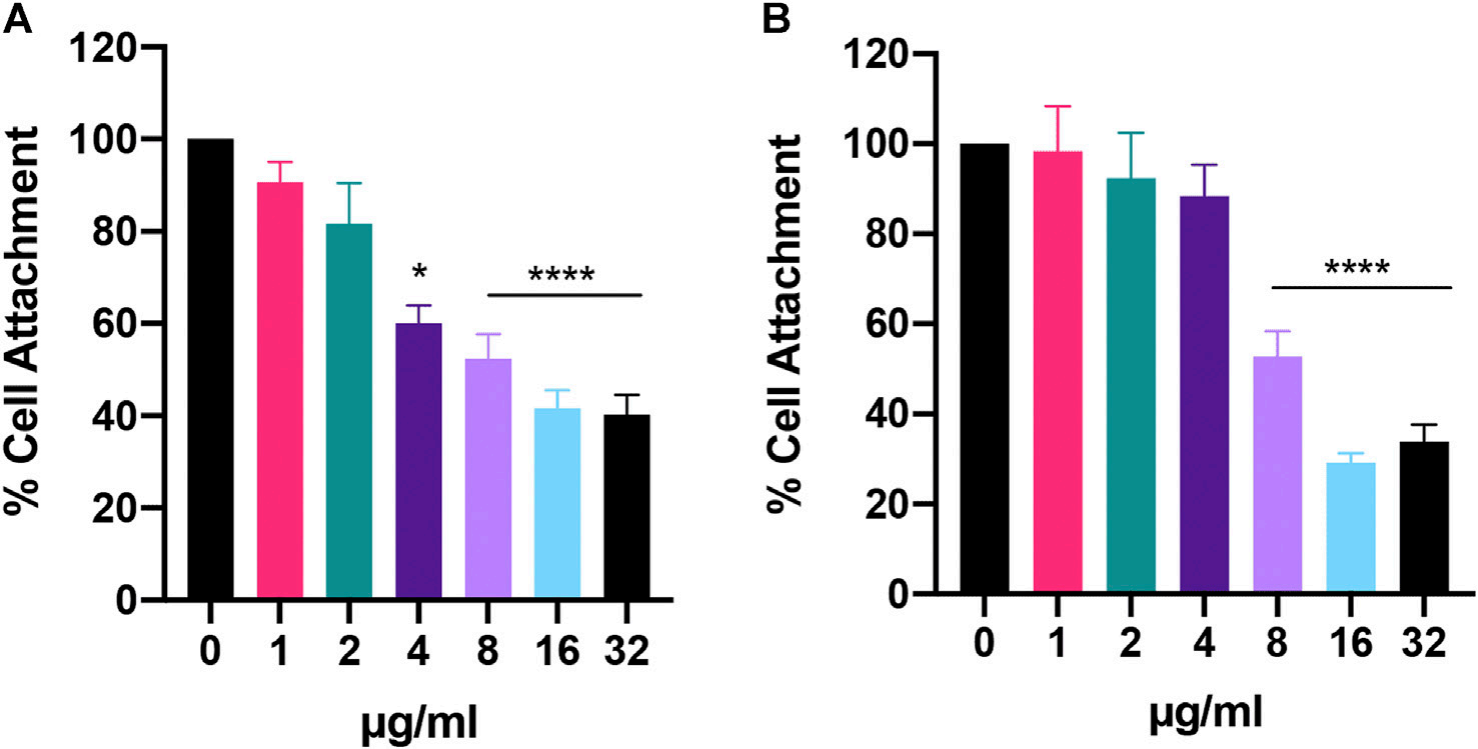
Bay 11-7085 shows anti-biofilm activity against *C. albicans* and *C. auris* initial cell attachment. **(A)** *C. auris* strain #0384 and **(B)** *C. albicans* strain SC5314 initial cell attachment was measured using XTT. X-axis represents concentrations of Bay 11-7085 in µg/ml. Statistical significance was determined using one-way ANOVA with Dunnett’s test for multiple comparisons. *p < 0.05; ****p < 0.0001. (*n* = 3, ±S.D.).

Lastly, when we looked at initial cell attachment, we found that Bay 11-7085 had greater activity against *C. auris* than *C. albicans* with >40% attachment inhibition at 4 μg/ml for *C. auris* (Figure 6A). At 8 μg/ml and 16–32 μg/ml *C auris* initial cell attachment was inhibited >40% and >50%, respectively (Figure 6A). While *C. albicans* was inhibited >40% and >60% at 8 μg/ml and 16–32 μg/ml, respectively (Figure 6B). In totality, we conclude that Bay 11-7085 can partially inhibit and eradicate both *C. albicans* and *C. auris* biofilm as well as hinder initial cell attachment.

Given that Bay 11-7085 is fungicidal at high concentrations (Figure 2), concentrations which showed anti-biofilm activity (Figures 5, 6), we hypothesized that Bay 11-7085 would hinder initial cell attachment through direct killing of biofilm-forming cells. To test this, we treated ∼ 10^6^ CFU/ml stationary-phase fungal cells with various concentrations of Bay 11-7085 for 90 min and measured their viability. As expected, our results showed that Bay 11-7085 is able to kill *C. albicans* and *C. auris* cells within 90 min of treatment (Supplementary Figure S1) which we conclude leads to the decrease of initial cell attachment.

### Bay 11-7085 has Anti-Biofilm Activity Toward *Staphylococcal*-*Candida* Polymicrobial Biofilm

**FIGURE 7:**
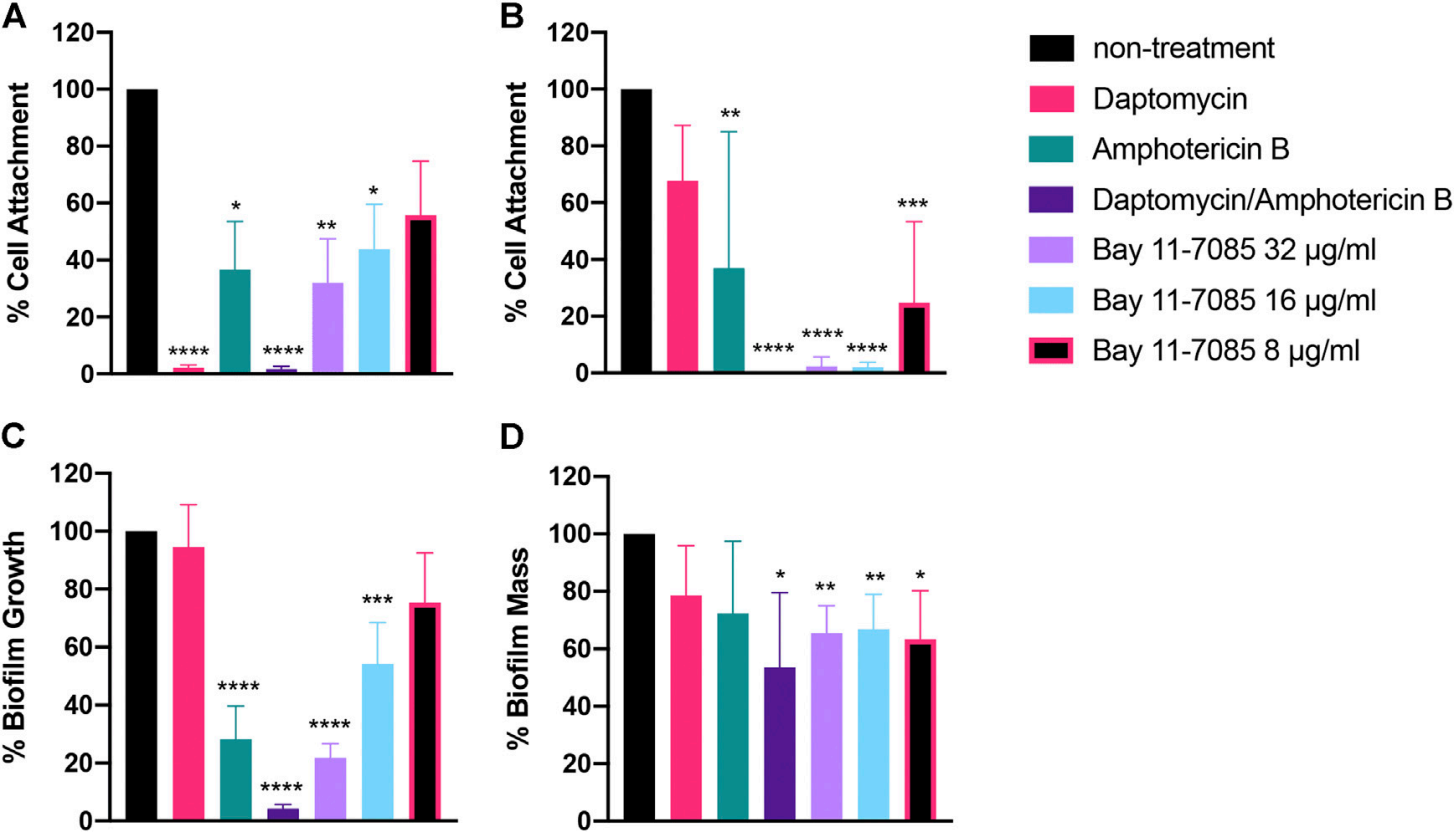
Bay 11-7085 shows anti-biofilm activity towards three phases of *S. aureus-C. albicans* polymicrobial biofilm maturation. Activity of Bay 11-7085 at various concentrations was tested against *S. aureus* VRS1-*C. albicans* SC5314 polymicrobial biofilm initial cell attachment, maturation, and eradication. *S. aureus* strain VRS1 and *C. albicans* strain SC5314 were grown overnight in BHI or YPD medium respectively. Cells were washed and co-cultured at a 1:10 ratio *S. aureus* strain VRS1 to *C. albicans* strain SC5413 under treatment of daptomycin 8× MIC (8 μg/ml), amphotericin B 32× MIC (16 μg/ml), a combination of both daptomycin and amphotericin B and Bay 11-7085 at 36, 16, and 8 μg/ml. Percent cell attachment was normalized to non-treatment control for **(A)** *S. aureus* strain VRS1, and **(B)** *C. albicans* strain SC5413 after treatment. Bay 11-7085s activity on *S. aureus* VRS1-*C. albicans* SC5314 biofilm inhibition **(C)** and eradication **(D)** of mature biofilm was measured using 1% CV. Statistical significance was determined using one-way ANOVA with Dunnett’s test for multiple comparisons **(A–C)** or Unpaired *t*-test **(D)**. *p < 0.05; **p < 0.01; ***p < 0.001; ****p < 0.000.1. (*n* = 3, ±S.D.).

So far, we showed Bay 11-7085 demonstrates promising activity on both *S. aureus* and *Candida spp.* monoculture biofilms (Figures 4–6). We thus hypothesized that Bay 11-7085 would also have potency on biofilm of a polymicrobial culture of *S. aureus* strain VRS1 and *C. albicans* strain SC5413. To test this hypothesis, we first verified inhibition of initial cell attachment of co-cultured *S. aureus* and *C. albicans* in 50% serum-BHI medium for 90 min on nitrocellulose disks. Disks were washed, sonicated, and plated on selective plates to enumerate the total CFU/membrane of *S. aureus* strain VRS1 and *C. albicans* strain SC5314 cells. Like monoculture experiments, Bay 11-7085 is potent enough to inhibit initial cell attachment of both *S. aureus* and *C. albicans* during polymicrobial co-culture. With *S. aureus* strain VRS1 showing a significant reduction of >55% at 16 μg/ml and >70% at 32 μg/ml (Figure 7A). When we analyzed *C. albicans* we find that Bay 11-7085 is consistent with our monoculture results and is more effective on *C. albicans* than *S. aureus*, with *C. albicans* being significantly reduced by >75% at 8 μg/ml and >97% starting at 16–32 μg/ml (Figure 7B).

We then questioned whether Bay 11-7085s effect on initial cell attachment would also hinder the maturation of polymicrobial *S. aureus-C. albicans* biofilm. To investigate this, we treated *S. aureus-C. albicans* co-cultures with Bay 11-7085 at various concentrations and quantified total biomass produced using 1% CV after 24 h of incubation. As expected, Bay 11-7085 was able to inhibit biofilm formation with >45% inhibition at 16 μg/ml and >75% inhibition at 32 μg/ml (Figure 7C).

Lastly, we hypothesized that Bay 11-7085 would have activity on mature *S. aureus-C. albicans* polymicrobial biofilm. We treated fully mature biofilm with various concentrations of Bay 11-7085 and quantified total biofilm remaining, using 1% CV, after a 24 h incubation period. Our findings showed that Bay 11-7085 can significantly eradicate >35% of mature biofilm at concentrations 8–32 μg/ml (Figure 7D). This reduction was greater than with antibiotic daptomycin (8× MIC) or antifungal amphotericin B (32× MIC) treatment alone and was not significantly different than treatment with a combination of both daptomycin and amphotericin B (Figure 7D). From these studies, we conclude that Bay 11-7085 is not only a potent agent against monoculture biofilms but is also effective at combating polymicrobial biofilms as well.

### Bay 11–7085 Shows Activity Against *C. albicans* in an *In Vitro* Multispecies Lubbock Chronic Wound Model

**FIGURE 8:**
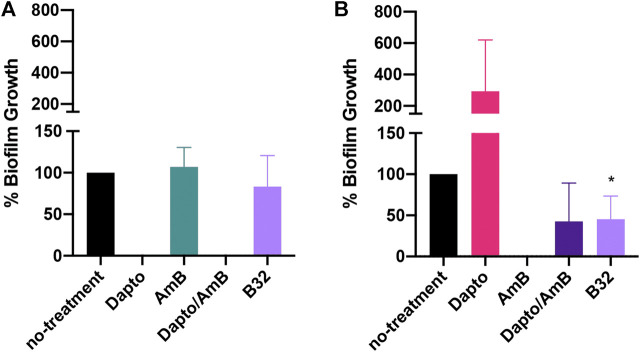
Bay 11-7085 inhibits *C. albicans* but not *S. aureus* growth in a multispecies Lubbock chronic wound model. **(A)** *S. aureus* strain VRS1 percent growth after treatment with Bay 11-7085 at 8 μg/ml (B8), 16 μg/ml (B16), or 32 μg/ml (B32) for 24 h. Treatment with 8× daptomycin (8 μg/ml, Dapto), 16× amphotericin B (16 μg/ml, AmB), or a combination of 8× daptomycin and 16× amphotericin B (Dapto/AmB) where used as controls. **(B)** *C. albicans* percent growth after treatment with Bay 11-7085 for 24 h. Statistical significance was determined using Unpaired *t*-test. *p < 0.05. (*n* = 3, ±S.D.).

We then asked whether Bay 11-7085 would have activity on *S. aureus* strain VRS1 or *C. albicans* strain SC5413 co-culture in a more clinically relevant *in vitro* wound like model. To assess this, we employed the use of the *in vitro* multispecies Lubbock chronic wound biofilm model originally designed by Sun et al. (2008). This model leveraged the use of a unique wound like media (WLM) composed of 50% Bolton Broth, 45% heparinized calf serum, and 5% laked horse blood to simulate a wound like environment. In WLM, *S. aureus* coagulase-positive strains will form a jelly-like mass of the media. For this reason, we decided to only test Bay 11-7085s activity against biofilm inhibition. Although Bay 11-7085 did not have any effect on *S. aureus* biofilm growth in WLM (Figure 8A), Bay 11-7085 was able to significantly reduce total CFU/ml of *C. albicans* residing within the polymicrobial biofilm by >50%, which was not significantly different than treatment with the combination of the antibiotic daptomycin and antifungal amphotericin B (Figure 8B). Interestingly, we found that treatment of daptomycin alone abolished *S. aureus* cells completely. However, treatment with daptomycin also allowed *C. albicans* to overgrow >200% more than no treatment control, while treatment with Bay 11-7085 did not allow overgrowth of either organism (Figure 8). We thus conclude that Bay 11-7085 has greater activity on *C. albicans* in an *in vitro* chronic wound biofilm model.

### Bay 11-7085 Shows Activity in a *C. elegans-C. albicans* Infection Model

**FIGURE 9:**
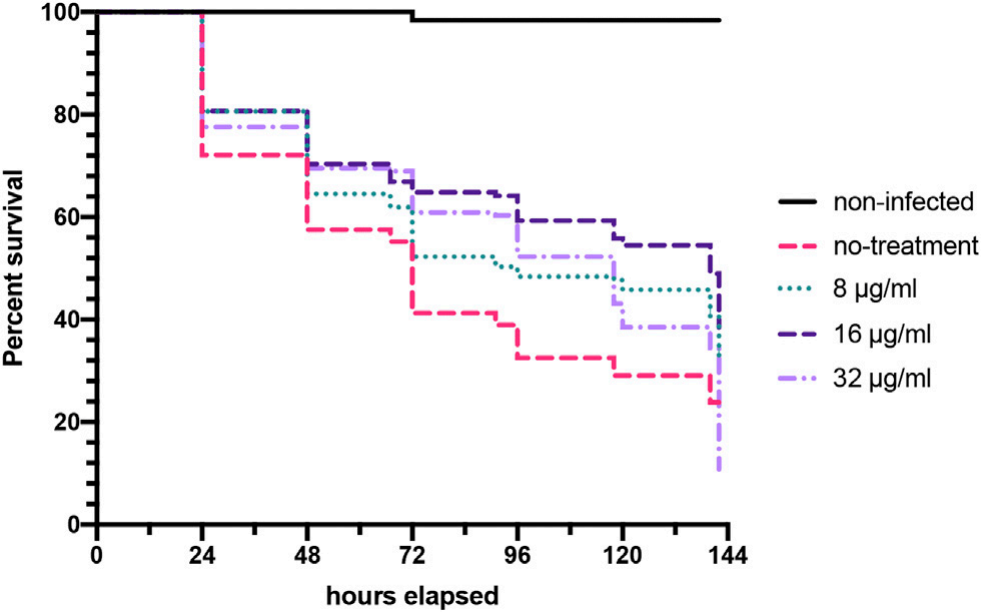
Bay 11-7085 prolongs *C. elegans* survival after *C. albicans* infection. *C. elegan* were exposed to *C. albicans* strain SC5314 grown on BHI agar plates, washed, and treated with various concentrations of Bay 11-7085 in liquid medium. Statistical significance was determined using Log-rank (Mantel-Cox). (*n* = 3).

Consistently our data has shown that Bay 11-7085 is a slightly more potent antimicrobial to *Candida* spp. rather than *S. aureus.* Therefore, to further evaluate Bay 11-7085 as a potential lead anti-infective compound against *Candida* spp. we wanted to test Bay 11-7085s potency in a whole animal infection model system. Accordingly, we tested whether Bay 11-7085 would be able to prolong or save the life of *C. elegans* during a *C. albicans* infection. Worms where first exposed to *C. albicans* strain SC5314 previously grown overnight on BHI agar plates. Worms were then washed and then treated with various concentrations of Bay 11-7085 for a total of 6 days. In the presence of Bay 11-7085, we found a significant increase in the survival of *C. elegans* starting at 8 μg/ml and up to 16 μg/ml, and although there was a significant increase in survival at 32 μg/ml, this was less than at 16 μg/ml (Figure 9). We thus conclude that Bay 11-7085 has potency at combating a *C. albicans* infection in a whole animal system at 8–16 μg/ml. Yet, at higher concentrations such as 32 μg/ml, we theorize a decrease in survival is due to toxicity at long exposure times such as >5 days.

## Discussion

In this study, we report a new bioactivity of Bay 11-7085 as an antimicrobial, effective against two biofilm-forming pathogens associated with wound infections: *S. aureus* and *Candida* spp. We show that the MIC of this compound against MRSA and VRSA strains is 4 μg/ml, while the MIC to *C. albicans* and *C. auris* is 0.5–1 μg/ml. Importantly, the antimicrobial activity of Bay 11-7085 is active on both bacterial and fungal biofilm in monoculture and polymicrobial co-culture.

Intriguingly, Bay 11-7085 was originally identified as a potential anti-inflammatory agent by inhibiting the dissociation of IκB-*α* from NF-κB thus blocking TNFα-induced phosphorylation of IκB-α (Pierce et al., 1997). This inhibition of IκB-α phosphorylation by Bay 11-7085 is irreversible and shows a median inhibitory concentration of 10 µM (2.5 μg/ml) (Pierce et al., 1997). At the dose of 20 mg/kg, Bay 11-7085 also showed *in vivo* anti-inflammatory efficacy in the Carrageenan rat paw edema model and the rat adjuvant arthritis model (Pierce et al., 1997). In addition, Bay 11-7085 can also induce apoptosis of cancerous cells by inhibiting NF-κB signaling. However, previous reports also show that Bay 11–7085 demonstrates anti-cancer activity in an NF-κB -independent manner, all of which indicates that Bay 11-7085 may possess multiple bioactivities (Azevedo et al., 2015; Vasu Govardhana Reddy et al., 2004). Given Bay 11-7085s high toxicity to cancer and primary cells *in vitro* (Figure 3C) we theorize that this is mainly due to its multiple mechanisms of cell apoptosis. Bacteria, on the other hand, do not have NF-κB signaling. Although yeasts have a similar but primitive NF-κB-like signaling pathway, called retrograde response (RTG) pathway, the inhibition of RTG in yeast does not result in the rapid killing of yeast cells (Srinivasan et al., 2010). Like ciprofloxacin, if Bay 11-7085 had only one target in MRSA, a resistant mutant would emerge during a 25-day serial passage (Friedman et al., 2006). However, according to our results this is not the cause (Figure 1D). Furthermore, membrane-active agents generally show low probability for resistance development (Kim et al., 2018a; Kim et al., 2018b; Kim et al., 2020). However, we observed that Bay 11-7085 did not affect membrane permeability (data not shown). Therefore, it is most likely that Bay 11-7085 may have multiple targets responsible for its antimicrobial activity.

Several reports, especially among immunocompromised patients, have associated antibacterial therapy with subsequent fungal infections (Azevedo et al., 2015). These fungal infections are believed to be caused by antibacterial agents killing microbial competitors of fungi (Azevedo et al., 2015), which our *in vitro* biofilm wound like model data also seemed to support (Figure 8B). To prevent this secondary mycoses, antifungal agents are used prophylactically during antibacterial treatments (Azevedo et al., 2015). Therefore, compounds combining narrow-spectrum antibacterial activity and antifungal potency can be clinically beneficial. To date, several agents with dual antibacterial and antifungal potency have been identified and synthesized (Vasu Govardhana Reddy et al., 2004; Ezabadi et al., 2008; Rubinchik et al., 2009; Zhang and Zhou, 2011; Vestergaard and Ingmer, 2019). However, except for the synthetic antimicrobial peptide omiganan, none of them are potent against biofilms, and although omiganan shows antibacterial potency against MRSA biofilms, it shows low antifungal activity against *C. albicans* with MIC of >64 μg/ml (Rubinchik et al., 2009). Bay 11-7085, however, has strong dual antimicrobial activity with a MIC of 0.5–4 μg/ml against *S. aureus* and *C. albicans* (Table 1, Supplementary Table S2) and is potent against biofilms formed by *S. aureus* or *C. albicans* (Figures 4–6). Furthermore, Bay 11-7085 shows narrow spectrum antibacterial activity to *S. aureus* over *E. faecium, E. faecalis,* and Gram-negative bacteria (Table 1). With its low probability of resistance development, Bay 11-7085 is a promising lead compound for prophylactic use.

Biofilms are composed of an ECM which house low metabolically active bacteria, facilitate quorum sensing, and thus create increased virulence to their resident bacteria relative to planktonic state bacteria (Kruppa, 2009; Nobile and Johnson, 2015; Cavalheiro and Teixeira, 2018). From our studies, we show that Bay 11-7085 can directly kill cells residing within both *S. aureus* and *Candida* spp. biofilm demonstrating that Bay 11-7085 is able to penetrate the ECM effectively. In addition, we show that *C. albicans* initial cell attachment is inhibited by Bay 11-7085s fungicidal effect. In the case of *S. aureus,* our data show that Bay 11-7085 cannot directly kill bacteria during the initial cell attachment stage of biofilm formation. Therefore, we theorize Bay 11-7085 may also have anti-virulence activity, possibly targeting adhesion molecules needed for biofilm attachment (Moormeier and Bayles, 2017). Furthermore, our data show that Bay 11-7085 was unable to decrease *C. albicans* cell viability within mature biofilm but was able to decrease total biomass. We hypothesis that this may be due to reduced virulence factors, similar to *S. aureus*, but which may affect the formation of ECM instead of only attachment. Although the activity of Bay 11-7085 on *C. auris* initially seems contradictory our data does show a trend where Bay 11-7085 was more effective at reducing total biomass than direct killing of biofilm residing cells. We therefore see Bay 11-7085 having dual functionality where it can kill biofilm residing cells directly but can independently affect virulence factors. All together, these data highlight Bay 11-7085 as a potent anti-biofilm agent capable of combating *S. aureus* and *Candida* spp. biofilm *in vitro.*

As stated previously, *S. aureus* and *C. albicans* are human commensals able to coexist together in various human organs such as the skin (Peleg et al., 2010; Kühbacher et al., 2017). However, both can readily turn from commensal to pathogenic when host immunity is compromised (Carolus et al., 2019). *C. albicans* and *S. aureus* have been co-isolated from a range of biofilm-associated infections, including cystic fibrosis, catheter-associated urinary tract infections, and burn wound infections (Peleg et al., 2010). These polymicrobial infections caused by *S. aureus* and *C. albicans* are difficult-to-treat due to formation of mixed biofilms and are more virulent due to so-called synergistic pathogen-pathogen interactions (Peleg et al., 2010; Carolus et al., 2019). While *S. aureus* is a weak biofilm former in the presence of serum, *C. albicans* can readily form biofilms. Once *C. albicans* biofilm matures, *S. aureus* can attach and form biofilms on the surface of *C. albicans* extracellular walls (Harriott and Noverr, 2009). *C. albicans* hyphae can pierce epithelial layers in this mixed biofilm, which facilitate *S. aureus* invasion (Peters et al., 2010). Moreover, *C. albicans* is known to enhance the production of *S. aureus* virulence factors, such as alpha- and delta-toxins (Todd and Peters, 2019). Consistently, it has been reported that mice co-infected with sub-lethal doses of *S. aureus* and *C. albicans* induce the rapid rise of morbidity and mortality (Carolus et al., 2019; Todd and Peters, 2019).

Considering the mechanisms of polymicrobial biofilm formation by *S. aureus* and *C. albicans*, Bay 11–7085 shows promising potential to inhibit the formation of these polymicrobial biofilms. Bay 11-7085 demonstrates relatively higher anti-biofilm activity against *Candida* spp. monoculture biofilms compared to *S. aureus* monoculture biofilms (Figures 4–6) and in addition shows a significant reduction in initial cell attachment, maturation, and eradication in a polymicrobial biofilm culture (Figure 7). It blocks fungal biofilm formation by direct killing of cells and prevents bacterial biofilm formation by inhibiting initial attachment. In physiological conditions, the key event for developing the polymicrobial biofilms is the maturation of *Candida* spp. Therefore, the fungicidal activity of Bay 11–7085 would effectively ruin this critical step and thus may impede the following step of *S. aureus* initial cell attachment. Although we were not able to show *S. aureus* reduction in our *in vitro* chronic wound like model, we did find that Bay 11-7085 was able to reduce *C. albicans* cell viability significantly. However, due to our experimental model, we were unable to quantify total biofilm mass. With the reduction of *C. albicans* cell viability we hypothesis that a Bay 11-7085 may possibly have greater effect on biofilm mass rather than cell viability which we are unable to test via our model. Further studies would be needed to assess biofilm mass reduction in a wound like model.

Regarding *S. aureus* and the lack of activity by Bay 11-7085 on our *in vitro* WLM model, we speculate this may be partially attributed to coagulation. More specifically, in our model, we used 5% laked horse blood to mimic a more relevant physiological environment. *S. aureus* strain VRS1 is coagulase-positive (CoP). In the presence of blood, *S. aureus* CoP strains secret coagulase enzymes, which aid in converting fibrinogen into fibrin. Bacteria begin to interlock and form blood clots. These blood clots are protective to the bacteria and have been shown to increase antimicrobial resistance (Giedrys-Kalemba et al., 2018). However, not all *S. aureus* strains are CoP and it is possible that Bay 11-7085 may have greater efficacy on *S. aureus* coagulate-negative strains, which would also correlate with our polymicrobial anti-biofilm data using blood-negative media (Figures 4, 7). However, additional studies would be needed to confirm the difference between other non-CoP strains.

Although Bay 11-7085 has advantages as an antimicrobial lead compound, it has limitations that need to be resolved. First, Bay 11-7085 shows cytotoxicity to cancerous cell lines and primary renal proximal tubule epithelial cells (RPTEC) (Figure 3C). Although Bay 11-7085 induces cancer cell apoptosis by inhibiting NF-κB signaling, its cytotoxicity to RPTEC is still unanswerable. In contrast to our *in vitro* toxicity results (Figure 3C), Bay 11-7085 did not cause observable toxicity in a rat model at the dose of 50 mg/kg (Pierce et al., 1997), which is consistent with our *C. elegans in vivo* toxicity results (Figure 3A). In any case, the cytotoxicity profile of Bay 11-7085 needs to be further evaluated. Furthermore, to repurpose Bay 11-7085 as an antimicrobial, its NF-κB inhibitory activity would also need to be reduced or nullified. Second, its antimicrobial mechanism should be elucidated for further optimization. Since Bay 11-7085 is expected to have multiple targets, it may be difficult to find its modes of action. However, current advances in deep learning based prediction, quantitative imaging, proteomic, genetic, and metabolomic analyses enable elucidating antimicrobial mechanisms having uncommon or multiple modes of action (Martin et al., 2020).

In conclusion, our studies show that Bay 11-7085 is a potent antimicrobial against MRSA, VRSA, and *Candida* spp. Bay 11-7085 shows anti-biofilm activity on both monocultures as well as in mixed cultures and does not show resistance after 25 days of serial passaging. We thus find Bay 11-7085 to be a promising lead compound useful for identifying new antimicrobial targets that show low resistance and anti-biofilm activity against pathogens commonly associated with wound biofilms.

## Data Availability

The raw data supporting the conclusion of this article will be made available by the authors, without undue reservation.
