# Supplementary material for: Repurposing Kinase Inhibitor Bay 11-7085 to Combat Staphylococcus aureus and Candida albicans Biofilms
Source: Front Pharmacol. 2021 May 5;12:675300. doi: 10.3389/fphar.2021.675300 (PMC8133364; doi:10.3389/fphar.2021.675300)
Supplement: Supplementary file 1 [file DataSheet1.pdf]

Table S1. Microbial strains used in this study.

Species	Strain	Genotype	Reference
Staphylococcus aureus	MW2	*SCCmec* Type IV	(Baba et al., 2002)
Staphylococcus aureus	LAC	*SCCmec* Type IVa	(Kennedy et al., 2008)
Staphylococcus aureus	WKZ-1	*SdrC, SdrD, Bbp,* SaPI2	(Bloemendaal et al., 2010)
Staphylococcus aureus	WKZ-2	*SCCmec, SdrC, SdrD, Bbp,* SaPI2	(Bloemendaal et al., 2010)
Staphylococcus aureus	ATCC 25923	Reference strain, *SCCmec*-like element lacking *mecA*	(Ito et al., 2001)
Staphylococcus aureus	ATCC 29213	Reference strain, *mecA* negative	(Ikonomidis et al., 2008), (Soni et al., 2015)
Staphylococcus aureus	ATCC 43300	*SCCmec* Type II, *pvl* negative	ATCC® 43300
Staphylococcus aureus	VRS1	*SCCmec, vanA*	(Bozdogan et al., 2004)
Enterococcus faecium	E007	Clinical Isolate from Massachusetts General Hospital, tetracycline resistant	(Garsin et al., 2001)
Enterococcus faecalis	MMH 594	Clinical isolate from University of Wisconsin and Clinics, resistance to erythromycin and gentamicin	(Huycke et al., 1991)
Klebsiella pneumoniae	KCTC2242	Reference strain	(Shin et al., 2012b)
Klebsiella pneumoniae	WGLW2	Reference strain, Broad Institute	Project accession: AMLM00000000
Pseudomonas aeruginosa	ATCC 27853	Reference Strain	ATCC® 27853, (Cao et al., 2017)
Pseudomonas aeruginosa	PA14	Reference Strain	(Rahme et al., 1995)
Enterobacter aerogenes	KCTC 2190	Reference Strain	(Shin et al., 2012a)

Candida albicans	SC5314	Reference Strain	(Bartelli et al., 2018)
Candida glabrata	ATCC 90030	Reference Strain	ATCC® 90030
Candida parapsilosis	ATCC 22019	Reference Strain	ATCC® 22019
Candida auris	AR-BANK #0381	Clinical isolate, fluconazole resistant	CDC Clinical Isolate Bank, Panel ID: CAU
Candida auris	AR-BANK #0382	Clinical isolate, fluconazole resistant	CDC Clinical Isolate Bank, Panel ID: CAU
Candida auris	AR-BANK #0383	Clinical isolate, fluconazole resistant	CDC Clinical Isolate Bank, Panel ID: CAU
Candida auris	AR-BANK #0384	Clinical isolate, fluconazole resistant	CDC Clinical Isolate Bank, Panel ID: CAU
Candida auris	AR-BANK #0385	Clinical isolate, fluconazole resistant	CDC Clinical Isolate Bank, Panel ID: CAU
Candida auris	AR-BANK #0386	Clinical isolate, fluconazole resistant	CDC Clinical Isolate Bank, Panel ID: CAU
Candida auris	AR-BANK #0387	Clinical isolate, fluconazole resistant	CDC Clinical Isolate Bank, Panel ID: CAU
Candida auris	AR-BANK #0388	Clinical isolate, fluconazole resistant	CDC Clinical Isolate Bank, Panel ID: CAU
Candida auris	AR-BANK #0389	Clinical isolate, fluconazole resistant	CDC Clinical Isolate Bank, Panel ID: CAU
Candida auris	AR-BANK #0390	Clinical isolate, fluconazole resistant	CDC Clinical Isolate Bank, Panel ID: CAU

Table S2. Minimum inhibitory concentration (μg/ml) of Bay 11-7085 against *Candida auris* antifungal resistant clinical isolates.

Strain	Bay 11-7085	Amphotericin B
C. auris AR-BANK #0382 (CDC)	0.5	0.5
C. auris AR-BANK #0383 (CDC)	1	1
C. auris AR-BANK #0384 (CDC)	1	1
C. auris AR-BANK #0385 (CDC)	1	2
C. auris AR-BANK #0386 (CDC)	1	2
C. auris AR-BANK #0387 (CDC)	1	2
C. auris AR-BANK #0388 (CDC)	1	1
C. auris AR-BANK #0389 (CDC)	0.5	2
C. auris AR-BANK #0390 (CDC)	1	2

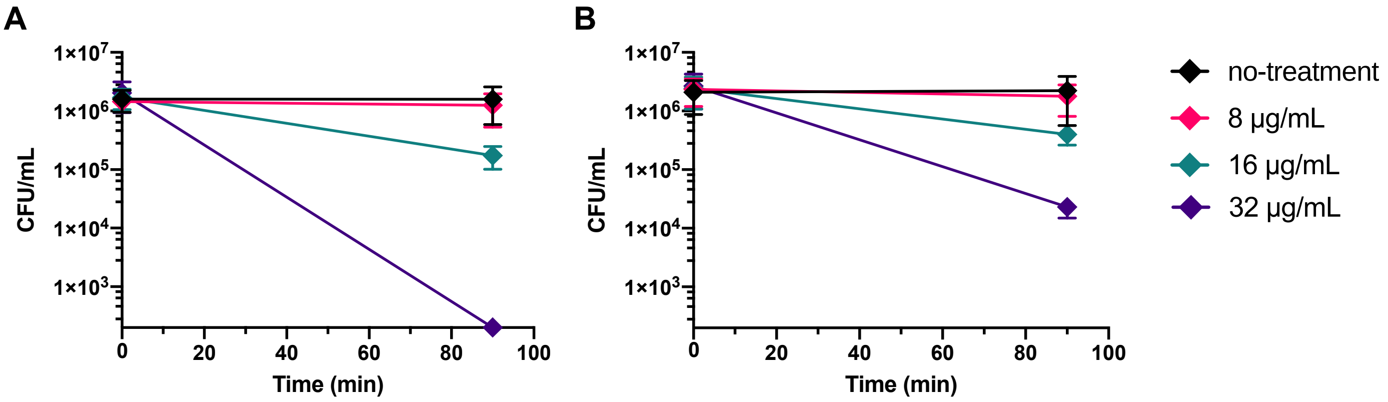

Figure S1. Bay 11-7085 inhibits *C. albicans* and *C. auris* initial cell attachment through direct killing of yeast cells. CFU/ml of **(A)** *C. albicans* strain SC5314 and **(B)** *C. auris* strain #0834 after 90 min of treatment with multiply concentrations of Bay 11-7085. (n=3, ± S.D.)

References

Baba, T., Takeuchi, F., Kuroda, M., Yuzawa, H., Aoki, K., Oguchi, A., et al. (2002). Genome and virulence determinants of high virulence community-acquired MRSA. Lancet 359, 1819–1827. doi:10.1016/s0140-6736(02)08713-5.

Bartelli, T. F., Bruno, D. do C. F., and Briones, M. R. S. (2018). Whole-Genome Sequences and Annotation of the Opportunistic Pathogen Candida albicans Strain SC5314 Grown under Two Different Environmental Conditions. Genome Announc 6, e01475-17. doi:10.1128/genomea.01475-17.

Bloemendaal, A. L. A., Brouwer, E. C., and Fluit, A. C. (2010). Methicillin Resistance Transfer from Staphylocccus epidermidis to Methicillin-Susceptible Staphylococcus aureus in a Patient during Antibiotic Therapy. Plos One 5, e11841. doi:10.1371/journal.pone.0011841.

Bozdogan, B., Ednie, L., Credito, K., Kosowska, K., and Appelbaum, P. C. (2004). Derivatives of a Vancomycin-Resistant Staphylococcus aureus Strain Isolated at Hershey Medical Center. Antimicrob Agents Ch 48, 4762–4765. doi:10.1128/aac.48.12.4762-4765.2004.

Cao, H., Lai, Y., Bougouffa, S., Xu, Z., and Yan, A. (2017). Comparative genome and transcriptome analysis reveals distinctive surface characteristics and unique physiological potentials of Pseudomonas aeruginosa ATCC 27853. Bmc Genomics 18, 459. doi:10.1186/s12864-017-3842-z.

Garsin, D. A., Sifri, C. D., Mylonakis, E., Qin, X., Singh, K. V., Murray, B. E., et al. (2001). A simple model host for identifying Gram-positive virulence factors. Proc National Acad Sci 98, 10892–10897. doi:10.1073/pnas.191378698.

Huycke, M. M., Spiegel, C. A., and Gilmore, M. S. (1991). Bacteremia caused by hemolytic, high-level gentamicin-resistant Enterococcus faecalis. Antimicrob Agents Ch 35, 1626–1634. doi:10.1128/aac.35.8.1626.

Ikonomidis, A., Michail, G., Vasdeki, A., Labrou, M., Karavasilis, V., Stathopoulos, C., et al. (2008). In Vitro and In Vivo Evaluations of Oxacillin Efficiency against mecA-Positive Oxacillin-Susceptible Staphylococcus aureus ▿. Antimicrob Agents Ch 52, 3905–3908. doi:10.1128/aac.00653-08.

Ito, T., Katayama, Y., Asada, K., Mori, N., Tsutsumimoto, K., Tiensasitorn, C., et al. (2001). Structural Comparison of Three Types of Staphylococcal Cassette Chromosome mec Integrated in the Chromosome in Methicillin-Resistant Staphylococcus aureus. Antimicrob Agents Ch 45, 1323–1336. doi:10.1128/aac.45.5.1323-1336.2001.

Kennedy, A. D., Otto, M., Braughton, K. R., Whitney, A. R., Chen, L., Mathema, B., et al. (2008). Epidemic community-associated methicillin-resistant Staphylococcus aureus: Recent clonal expansion and diversification. Proc National Acad Sci 105, 1327–1332. doi:10.1073/pnas.0710217105.

Rahme, L. G., Stevens, E. J., Wolfort, S. F., Shao, J., Tompkins, R. G., and Ausubel, F. M. (1995). Common virulence factors for bacterial pathogenicity in plants and animals. Science (New York, N.Y.) 268, 1899–1902. doi:10.1126/science.7604262.

Shin, S. H., Kim, S., Kim, J. Y., Lee, S., Um, Y., Oh, M.-K., et al. (2012a). Complete Genome Sequence of Enterobacter aerogenes KCTC 2190. J Bacteriol 194, 2373–2374. doi:10.1128/jb.00028-12.

Shin, S. H., Kim, S., Kim, J. Y., Lee, S., Um, Y., Oh, M.-K., et al. (2012b). Complete Genome Sequence of the 2,3-Butanediol-Producing Klebsiella pneumoniae Strain KCTC 2242. J Bacteriol 194, 2736–2737. doi:10.1128/jb.00027-12.

Soni, I., Chakrapani, H., and Chopra, S. (2015). Draft Genome Sequence of Methicillin-Sensitive Staphylococcus aureus ATCC 29213. Genome Announc 3, e01095-15. doi:10.1128/genomea.01095-15.
